# Aberrant expression and hormonal regulation of Galectin-3 in endometriosis women with infertility

**DOI:** 10.1007/s40618-016-0435-7

**Published:** 2016-02-17

**Authors:** H. Yang, J. Yin, K. Ficarrotta, S. H. Hsu, W. Zhang, C. Cheng

**Affiliations:** Department of Reproductive Endocrinology, Obstetrics and Gynecology Hospital, Fudan University, 413 Zhaozhou Road, Shanghai, 200011 China; Department of Gynecology, Chongqing Ninth People’s Hospital, Chongqing, China; Department of Chemical and Biomedical Engineering, University of South Florida, Tampa, FL 33620 USA; Department of Medicine, SUNY Downstate Medical Center, New York, NY USA; Department of Genetics, Geisel School of Medicine at Dartmouth, Hanover, NH USA

**Keywords:** Endometriosis, Galectin-3 (Gal-3), Uterine receptivity, Sex hormone

## Abstract

**Objective:**

To investigate the role and potential molecular mechanism of Galectin-3 (Gal-3) in the etiology of endometriosis-associated infertility.

**Methods:**

We detected Gal-3 expression in eutopic endometrium from women with endometriosis-associated infertility and healthy women without endometriosis or infertility. We then evaluated Gal-3 expression in endometrial glandular epithelial cells (EECs) and endometrial stromal cells (ESCs) and investigated its response to hormone stimulation in EECs and ESCs from both groups of women.

**Results:**

Results of real-time PCR and western blot analysis showed Gal-3 expression in both proliferative and secretory stages of the menstrual cycle decreased significantly in women with endometriosis-associated infertility compared to healthy women. The changes in expression of Gal-3 were more dramatic in EECs than ESCs. Moreover, estrogen (E2) and progesterone (P4) induced Gal-3 expression in EECs of healthy groups, and P4 was more significant than E2 and combined E2 and P4 (E2P4). However, in the endometriosis group, P4 failed to induce a similar increase in Gal-3 expression.

**Conclusions:**

Our results suggest that aberrant expression of Gal-3 might contribute to infertility in patients with endometriosis due to progesterone resistance.

## Introduction

Endometriosis is a benign gynecological disorder with malignant biological characteristics and is defined by the presence of endometrial glands and stroma outside the uterine cavity. Ectopic endometrial cells can spread to pelvic organs such as rectum, bladder, and ovaries. This spreading contributes to various clinical manifestations including irregular uterine bleeding, dyspareunia, chronic pelvic pain, and infertility: a condition affecting 10 % of women of reproductive age [1–3]. Statistically, 30–50 % of females with endometriosis are infertile and 25–50 % of females with infertility have endometriosis [4]. Although the association between endometriosis and infertility has been well established, the underlying mechanisms remain unknown.

It has been proposed that abnormal endometrial development in patients with endometriosis contributes to endometriosis-related infertility [5]. It is well known that endometrial receptivity is vital in women’s fertility. Human tissue and mouse model studies have demonstrated that the molecular markers of endometrial receptivity are altered in both humans and mice with endometriosis. Integrins, matrix metalloproteinases (MMPs), and homeobox genes (HoxA10) display aberrant expression patterns in the eutopic endometrium of women with endometriosis [6, 7]. Other alterations of biochemical or molecular markers have also been reported, including changes in the levels of vascular endothelial growth factor (VEGF) [8] and interleukin 6 (IL-6) [9]. Galectin-3 (Gal-3), a β-galactoside-binding protein, has been related to endometrial receptivity during embryonic implantation recently [10, 11]. Gal-3 with a ~31 kDa lectin contains a collagen-α-like domain, an N-terminal domain, and the carbohydrate recognition domain (CRD) [12]. These three structures allow Gal-3 to possess specific biological functions, including cell adhesion, migration, cell-extracellular matrix interactions [13], immune response, [14] and signal transduction [15]. It has been shown that Gal-3 is expressed in many cell types, including endometrial cells and trophoblast cells [16–19]. Previous studies have reported that Gal-3 is specifically expressed in endometrial cells in the secretory phase [10, 20], in placental tissue during early pregnancy, and in decidua surrounding the site of implantation. Due to these findings, we speculated that the expression of Gal-3 might be aberrant in endometriosis patients with infertility.

Additionally, Gla-3 has been known as a component of a nuclear and cytoplasmic complex, shuttling between the nucleus and cytoplasm [21]. Estrogen and progesterone belong to the nuclear hormone superfamily transcription factors, which play important role in the formation of receptive endometrium. Furthermore, it has been demonstrated that endometriosis-related infertility is associated with abnormal sex hormone regulation [22]. Thus, it would be interesting to explore whether the expression of Gal-3 is regulated by sex hormones in secondary infertility of endometriosis patients.

## Materials and methods

### Tissue collection

We collected endometrial tissue samples from 34 women, aged 28–35 years (mean age 31.51 ± 3.52), who consulted for infertility and were found to have surgically and histologically-confirmed endometriosis. As controls, we also collected endometrial biopsy specimens from 34 healthy women aged 30–35 years (mean age 32.55 ± 3.90). Women in the control group underwent tubal sterilization and were laparoscopically confirmed to be free of endometriosis. These women displayed normal menstrual cycles and had not received anti-inflammatory or hormonal therapy within 3 months prior to surgery. The phase of the subjects’ menstrual cycle was determined according to the criteria of Noyes et al. [23].

The endometrial tissue samples from each group were digested for cell culture, snap frozen and kept under −80 °C for subsequent real-time PCR and western blot analysis, or fixed in 4 % paraformaldehyde for paraffin blocks. Informed consent was obtained in writing from all subjects before surgery.

### Cell culture

Primary proliferative phase endometrial stromal and glandular epithelial cells were obtained from six subjects in the endometriosis group and six subjects in the control group. For each subject, secretory phase endometrial stromal and glandular epithelial cells were also obtained. Endometrial tissue was transported from the site of collection to the laboratory in Hanks’ balanced salt solution. The tissue was then minced and digested in Hanks’ balanced salt solution containing 1 % penicillin, 1 % streptomycin, 5 % collagenase, and 0.5 % deoxyribonuclease at 37 °C for 30 min with agitation. The dispersed endometrial cells were separated by filtration through a wire sieve (73-mm diameter pore, Sigma). The endometrial glands (largely undispersed) were retained by the sieve, whereas the dispersed stromal cells passed through the sieve into the filtrate. Endometrial glandular epithelial cells (EECs) were plated on Madrigal coated 12 well-plates, while endometrial stromal cells (ESCs) were plated on plastic 12-well plates. Both types of cells were plated in DMEM/F12 phenol red medium (Gibco Invitrogen,Carlsbad, CA, USA) containing 10 % fetal bovine serum (FBS) (Gibco). Cell cultures were maintained at 37 °C in a humidified atmosphere (5 % CO_2_) and were allowed to replicate to confluence. Thereafter, cells were passaged by standard methods of trypsinization and allowed to replicate to confluence, which required approximately 24–48 h. Cells after first passage were characterized as described previously [24].

### Real-time PCR analysis

Total RNA was extracted from 34 samples from the endometriosis group and 34 samples from the control group using Trizol Reagent (Invitrogen). Total RNA (1 μg) was reverse transcribed using a PrimeScript RT reagent kit (Takara, Dalian, China). Reverse-transcription PCR was performed prior to quantitative real-time PCR. The mRNA levels were determined by real-time PCR using SYBR Premix Ex Taq (TaKaRa) with the Applied Biosystems 7000 system SDS software as previous described [25]. Glyceraldehyde 3-phosphate dehydrogenase (GAPDH) was used as an endogenous control to normalize for differences in the amount of total RNA in each sample. The primer sequences and the sizes of the amplified fragments were as follows: Gal-3 (93 bp) 5′-CTT CCA CTT TAA CCC ACG CTT CAA-3′ (sense), 5′-TGT CTT TCT TCC CTT CCC CAG TTA TT-3′ (anti-sense); GAPDH (131 bp) 5′-ATG ACC CCT TCA TTG ACC-3′ (sense), 5′-GAA GAT GGT GAT GGG ATT TC-3′ (anti-sense).

### Immunohistochemistry

The paraffin blocks were cut into sections of 4 μm, mounted on polylysine-coated microslides, dewaxed, and rehydrated. Then, tissue sections were incubated in 3 % hydrogen peroxide at room temperature. For antigen retrieval, tissue slides were immersed in citric acid and boiled in a microwave oven. The sections were washed in distilled water and phosphate buffered saline (PBS, Gibco), and then subjected to bovine serum to block unspecific binding agents. This step was followed by overnight exposure (4 °C) to the mouse anti-human Gal-3 antibody (mab1154, R&D Systems Inc., Minneapolis, MN, USA) in a humidified chamber. After being rinsed in PBS, the sections were incubated with the bridging rabbit anti-mouse immunoglobulins conjugated with horseradish peroxidase (HRP)-labelled dextran polymer for 1 h. After washing in PBS, diaminobenizidine tetrahydrochloride (DAB) solution was applied, followed by running tap water as well as nuclear counterstaining with haematoxylin (Sigma, USA). The stained slides were viewed under microscopy (OLYMPUS OPTICAL CO., Ltd.) under 400× magnification. Positive cells were characterized by the brown staining of Gal-3 antibody. The intensity and distribution of the staining reaction were evaluated by two blinded, independent observers.

### Western blot

Protein samples were extracted from tissues or cells, and the protein concentration was measured by bicinchoninic acid assay (BCA). Samples were run in SDS-PAGE gel, transferred to nitrocellulose membranes, and immunoblotted overnight with gentle shaking at 4 °C with primary antibodies. The primary antibodies included monoclonal mouse anti-human Gal-3 antibody (1:2500) and monoclonal mouse anti-human GAPDH (Kangchen, Shanghai, China) antibody (1:5000). This procedure was followed by incubation with horseradish peroxidase-conjugated secondary antibodies. The reactions were detected by enhanced chemiluminescence assay. GAPDH was used as an endogenous control for normalization.

### Hormonal stimulation protocol

For the hormone stimulation test, 5 × 10^5^ cells in 1 ml of media were plated on a 6-well plate and grown for 24 h in normal medium containing 10 % FBS without antibiotics. Then, the media were replaced by serum-free and phenol red-free culture media for 24 h to prepare for the stimulation experiment. Stimulation was performed under various concentrations of 17β-estradiol (E2, 0, 10^−10^–10^−6^ M, Sigma) alone and under various concentrations of progesterone (P4, 0, 10^−9^–10^−5^ M, Sigma) alone. The same experiment was performed with E2, P4, or E2 combined with P4 (E2P4, 10^−8^ M E2, 10^−7^ M P4, Sigma) for 24, 48, and 72 h. The control was plated in phenol red-free culture media containing charcoal-stripped 10 % FBS (Bioind, Shanghai, China).

### Statistical analysis

Data were expressed in terms of mean ± SEM. One-way ANOVA analyses were performed and least significant difference (LSD) tests were applied for post hoc testing using SPSS software version 15.0 with *p* < 0.05 considered statistically significant.

## Results

### Gal-3 expression in endometria of endometriosis and control groups

**Fig. 1:**
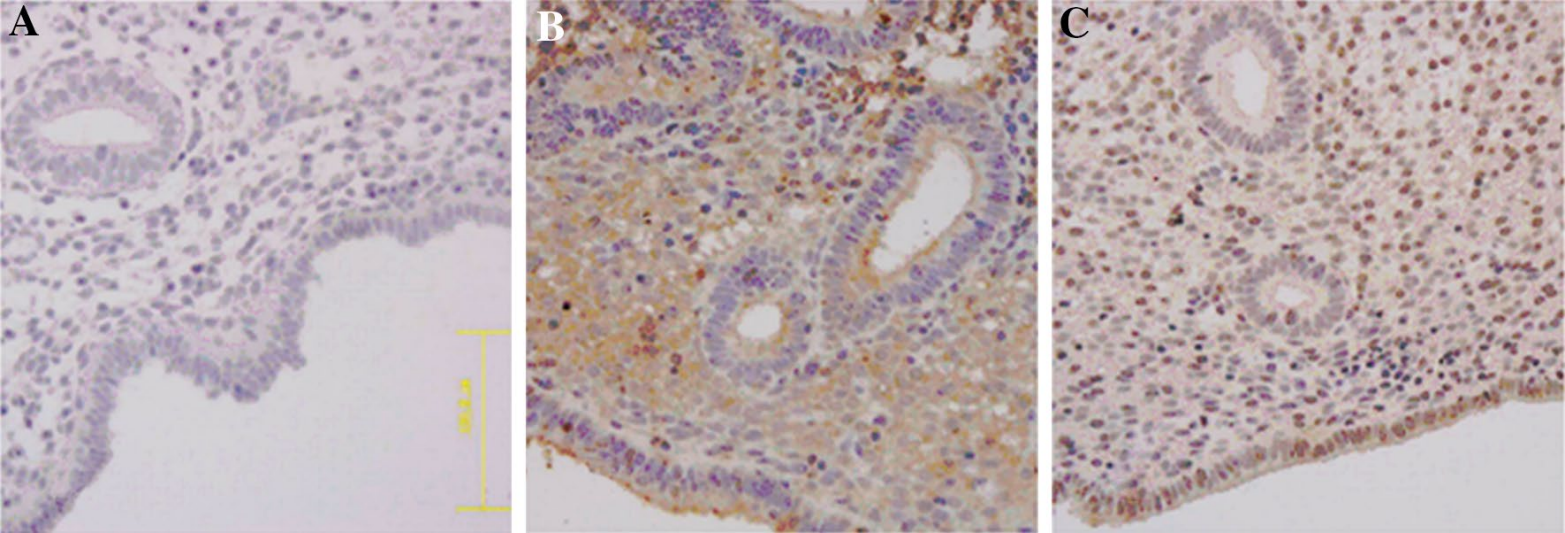
Immunohistochemistry analysis of Gal-3 expression. **a** Negative control. **b** Secretory phase endometrium from healthy controls. **c** Secretory phase eutopic endometrium from endometriosis patients with infertility. Mouse IgG was used in the negative control (**a**), while Gal-3 antibody was used other analyses (**b**, **c**)

To verify whether expression of Gal-3 is abnormal in the endometria of the endometriosis group, we performed immunochemistry analysis to display the expression pattern of Gal-3 proteins in endometria from both the healthy and endometriosis group (Fig. 1). Our results showed that Gal-3 was presented in the endometrium from both the control group (Fig. 1b) and the endometriosis group (Fig. 1c).

**Fig. 2:**
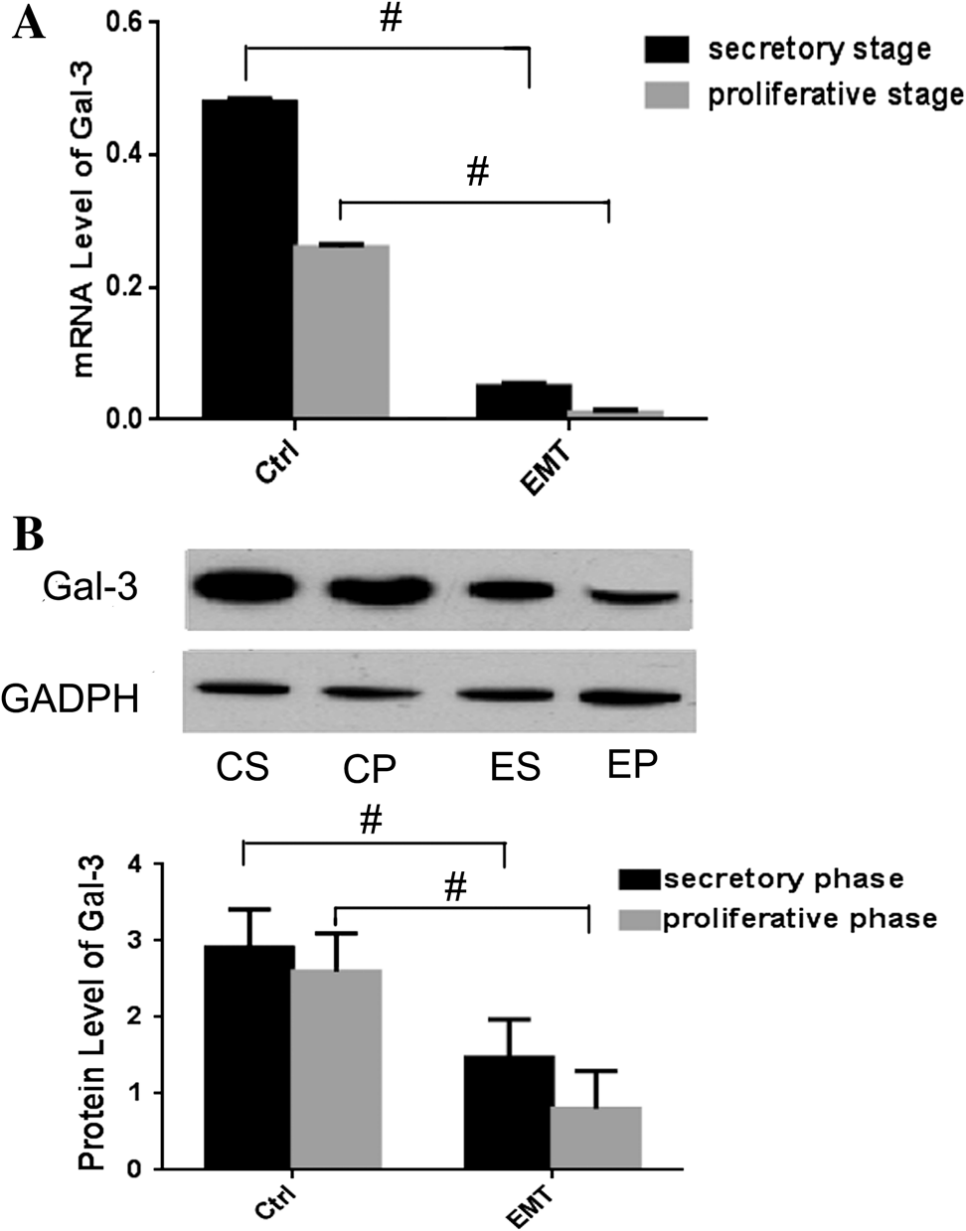
Gal-3 mRNA and protein expression in endometria samples from patients and healthy women. **a** Relative expression levels of Gal-3 mRNA in different samples measured by real-time PCR analysis. **b** Relative expression levels of Gal-3 protein in different samples measured by western blot analysis. Expression levels of Gal-3 were normalized against those of GAPDH in matched samples. *ES* endometria of patient with endometriosis in secretory phase, *EP* endometria of patient with endometriosis in proliferative phase, *CS* endometria of controls in secretory phase, *CP* endometria of controls in proliferative phase, *Control* control group, *EMT* endometriosis group; ^#^ *p* < 0.05

Then, Gal-3 mRNA expression levels in all endometrium samples were detected by real-time PCR analysis. As shown in Fig. 2a, Gal-3 showed significantly higher levels of expression in the secretory phase than the proliferative phase, regardless of group. Moreover, during both phases, Gal-3 expression was significantly down-regulated (secretory stage 0.47 ± 0.02 vs 0.05 ± 0.01, *p* < 0.05; proliferative phase 0.26 ± 0.02 vs 0.02 ± 0.01, *p* < 0.05) in eutopic endometrium from endometriosis patients compared to the normal endometrium. This result was confirmed at the protein level by western blot analysis (Fig. 2b).

### Gal-3 expression in epithelial cells and stromal cells of endometria

**Fig. 3:**
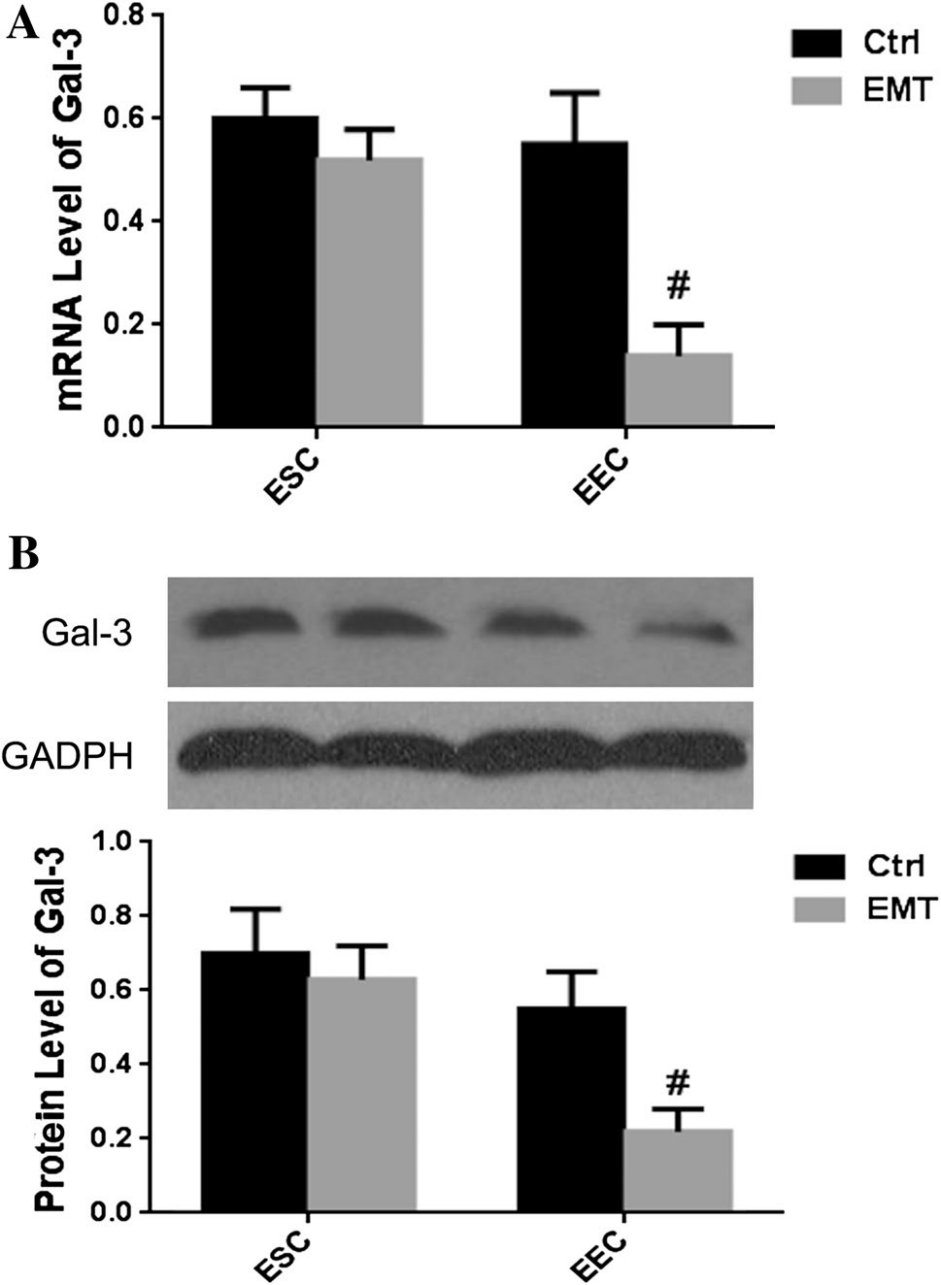
Expression of Gal-3 mRNA and protein in epithelial cells and stromal cells from secretory phase endometria. **a** Relative expression levels of Gal-3 mRNA in EECs and ESCs. **b** Relative expression levels of Gal-3 protein in EECs and ESCs; *Ctrl* control group, *EMT* endometriosis group, *EECs* endometrial glandular epithelial cells, *ESCs* endometrial stromal cells; ^#^ *p* < 0.05

The endometrium becomes receptive to embryonic attachment in the secretory phase of the menstrual cycle; thus, we explored whether Gal-3 expression is different in EECs and ESCs during this phase. The results of real-time PCR showed that Gal-3 expression was significantly down-regulated in EECs from the endometriosis group compared to the healthy group (0.14 ± 0.06 vs 0.59 ± 0.07, *p* < 0.05) (Fig. 3a). In contrast, there was no significant change in Gal-3 expression in ESCs from the control group compared to the endometriosis group; however, there was a trend of down-regulation of Gal-3 in the endometriosis group. We further confirmed these results by western blot analysis. As shown in Fig. 3b, a significant down-regulation of Gal-3 protein was observed in EECs but not ESCs of eutopic endometrial samples from the endometriosis group compared to the control group (0.22 ± 0.05 vs 0.58 ± 0.07, *p* < 0.05).

### Hormone-stimulated Gal-3 expression in endometrial epithelial cells

**Fig. 4:**
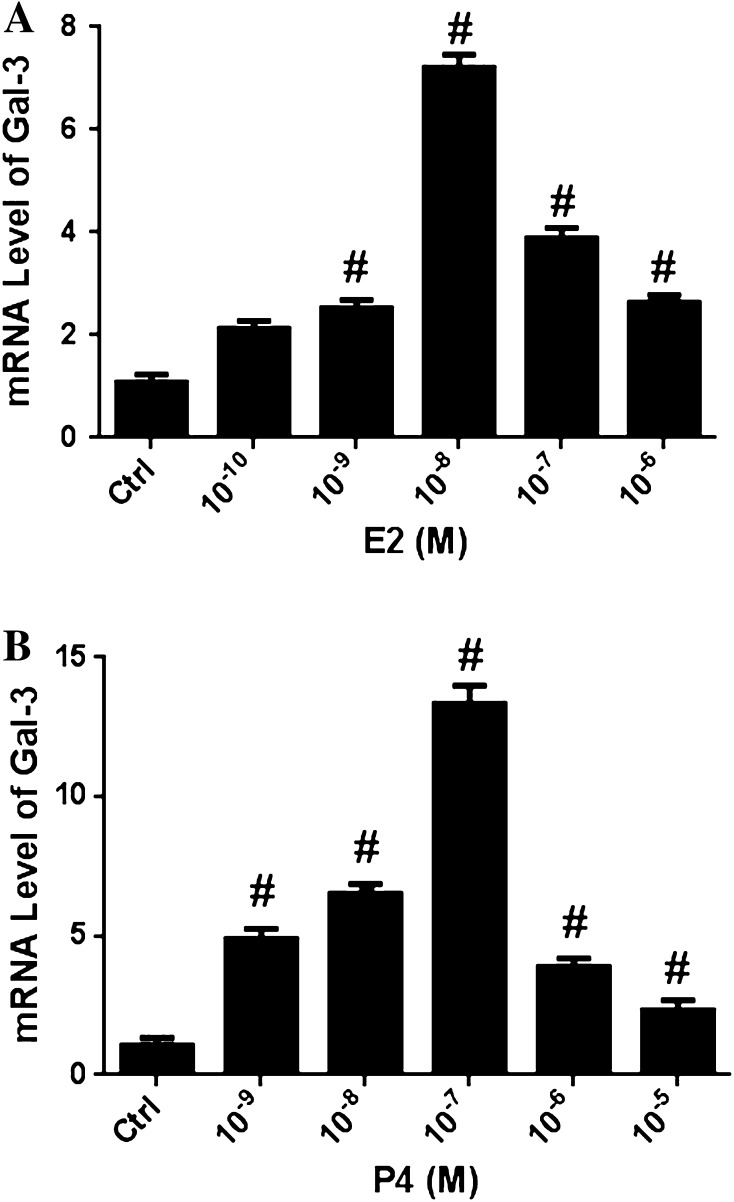
Hormonal regulation of Gal-3 expression by E2 and P4 in Endometrial epithelial cells. EECs were treated with E2 (**a**) (Ctrl, 10^−10^–10^−6^ M) and P4 (**b**) (Ctrl, 10^−9^–10^−5^M) for 24 h, mRNA level of Gal-3 was determined by real-time PCR. Gal-3 expression was analyzed by real-time PCR and GAPDH was used as an endogenous control to normalize for differences. *EECs* endometrial glandular epithelial cells; ^#^ *p* < *0.05*

To further investigate the hormone regulation of Gal-3 expression in EECs, various concentrations of E2 and P4 were used to co-culture EECs from the control group for 24 h before mRNA levels of Gal-3 were detected. Our results showed that Gal-3 was up-regulated by E2 and P4 at all concentrations, reaching peak level at 10^−8^ and 10^−7^ M, respectively (Fig. 4a, b) Thus, concentrations of 10^−8^ M of E2 and 10^−7^ M of P4 were used in the subsequent experiments.

**Fig. 5:**
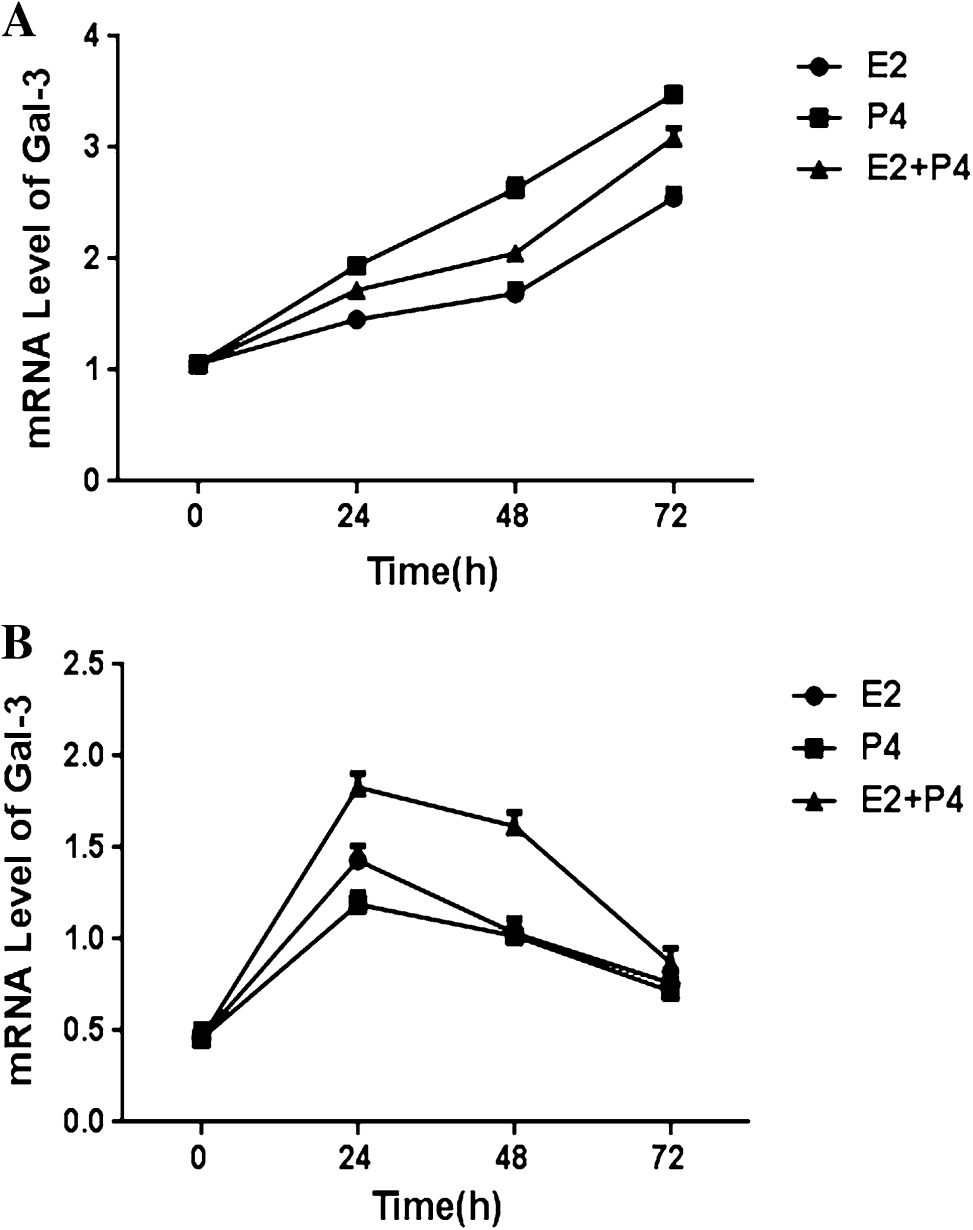
Defective progesterone regulation in endometrial epithelial cells from endometriosis women with infertility. EECs from control group (**a**) and endometriosis group (**b**) were treated with 10^−8^ M E2 with or without 10^−7^ M P4 and Gal-3 expression was analyzed at 0, 24, 48, 72 h. Gal-3 expression was analyzed by real-time PCR and GAPDH was used as an endogenous control to normalize for differences. *EECs* endometrial glandular epithelial cells; ^#^ *p* < *0.05*

Then, the expression of Gal-3 was detected in EECs pretreated with 10^−8^ M E2, 10^−7^ M P4, or 10^−8^ M E2 combined with 10^−7^ M P4 for 24, 48, and 72 h, respectively. In the control group, Gal-3 expression was induced by E2 or P4 treatment in a time dependent manner (Fig. 5a). Expression of Gal-3 in the E2P4 group was lower than that in the P4 treatment group and higher than that in the E2 treatment group. These results suggest Gal-3 induction is more sensitive to P4 than E2 treatment; however, in EECs from the eutopic endometria with endometriosis (Fig. 5b), Gal-3 expression increased slightly after hormone pretreatment for 24 h, and then decreased time-dependently. Furthermore, compared with the control group, the expression of Gal-3 was lower in the endometriosis groups at all time points.

## Discussion

Endometriosis shows a serious impact on female fertility, but the etiology and pathogenesis of endometriosis-related infertility are unknown. Thus, it is imperative to identify the molecular mechanism of endometriosis to develop an effective therapy for endometriosis patients with infertility. Several studies have reported that endometriosis is a major cause of infertility due to its adverse effect on endometrial receptivity to embryonic implantation [26].

Our previous study verified that Gal-3 plays an important role in the process of embryonic implantation [25]. Intracellular Gal-3 promoted proliferation and adhesion in endometrial cells. Decreased expression of Gal-3 hindered embryonic adhesion to endometrial epithelial cells and delayed proliferation of endometrial stromal cells in achieving optimal status to accommodate the invading embryo, resulting in failed embryonic implantation. Secreted Gal-3 inhibited cell proliferation and induced apoptosis of endometrial cells [27]. This study shows that Gal-3 is expressed in the endometrium of both endometriosis and healthy women, but is reduced significantly in the former. This suggests a defect in Gal-3 expression occurs in eutopic endometrium from endometriosis patients with infertility. Decreased Gal-3 expression in eutopic endometrium from patients with endometriosis may contribute to the defective formation of receptive endometrium, thus leading to infertility.

Hormonal regulation of cellular function impacts many dynamic biological changes occurring during the peri-implantation stage of the menstrual cycle. Estrogen and progesterone act coherently at certain time intervals to stimulate the expression of key molecules that regulate endometrial receptivity. Our results showed that Gal-3 expression specifically increased during the secretory phase of the menstrual cycle in both groups, indicating that Gal-3 may be regulated by sex hormones. To confirm this relationship, we investigated the effect of hormones on Gal-3 expression in both EECs and ESCs. We found that regulation of Gal-3 expression by E2 and P4 could be detected in EECs but not ESCs. This result indicates that Gal-3 may primarily contribute to the dynamic change of EECs during embryonic implantation. Then, we explored the physiological dose of E2 (10^−8^ M) and P4 (10^−7^ M) that maximized Gal-3 expression in EECs. The results suggested that Gal-3 is regulated by sex hormones, which is in agreement with our previous study [27]. In the control group, expression of Gal-3 was significantly increased when induced by P4 alone, compared to E2 alone or E2P4. From these results, we concluded that E2, to some degree, antagonized the increased expression of Gal-3 by P4 in normal endometrium. In the endometriosis group, E2 alone, P4 alone, nor E2P4 could increase Gal-3 expression to the level of the control group. This indicates that there is no induction of Gal-3 expression in response to E2 or P4 treatment in the endometriosis group. Previous reports have shown that progesterone resistance is one important factor for endometriosis. Moreover, Gal-3 can be detected in the peritoneal fluid of endometriosis patients [28–30]; thus, defective progesterone regulation in endometriosis women with infertility might account for decreased Gal-3 expression in eutopic endometrium.

In summary, we found decreased expression of Gal-3 in eutopic endometrium from endometriosis, which may account for the defective formation of receptive endometrium. We further showed that Gal-3 was regulated mainly by hormones in EECs. We also suggested that the failure of Gal-3 elevation by hormones in EECs from endometriosis patients may contribute to progesterone resistance in endometriosis-related infertility. Although our study preliminarily indicates that the defective expression of Gal-3 may contribute to infertility in patients with endometriosis, further research is needed to detail the pathways of Gal-3 in eutopic endometrium from endometriosis.
